# Right iliac fossa kidney: Differential diagnoses

**DOI:** 10.1002/ccr3.3724

**Published:** 2021-01-05

**Authors:** Daniel T. Doherty, Leszek Wolowczyk

**Affiliations:** ^1^ Manchester University Hospital NHS Foundation Trust Manchester UK; ^2^ Faculty of Biology Medicine & Health University of Manchester Manchester UK

**Keywords:** hernia, kidney, renal transplant, right iliac fossa

## Abstract

A right iliac fossa kidney is rarely encountered by the general clinician but multiple diagnoses should be considered.

## Graphical abstract

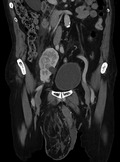

## 1 QUESTION

What are the differential diagnoses of a right iliac fossa kidney?

## 2 ANSWERS

1. Renal transplant
2. Supernumerary pelvic kidney
3. Descended kidney secondary to large inguinal hernia

**FIGURE 1:**
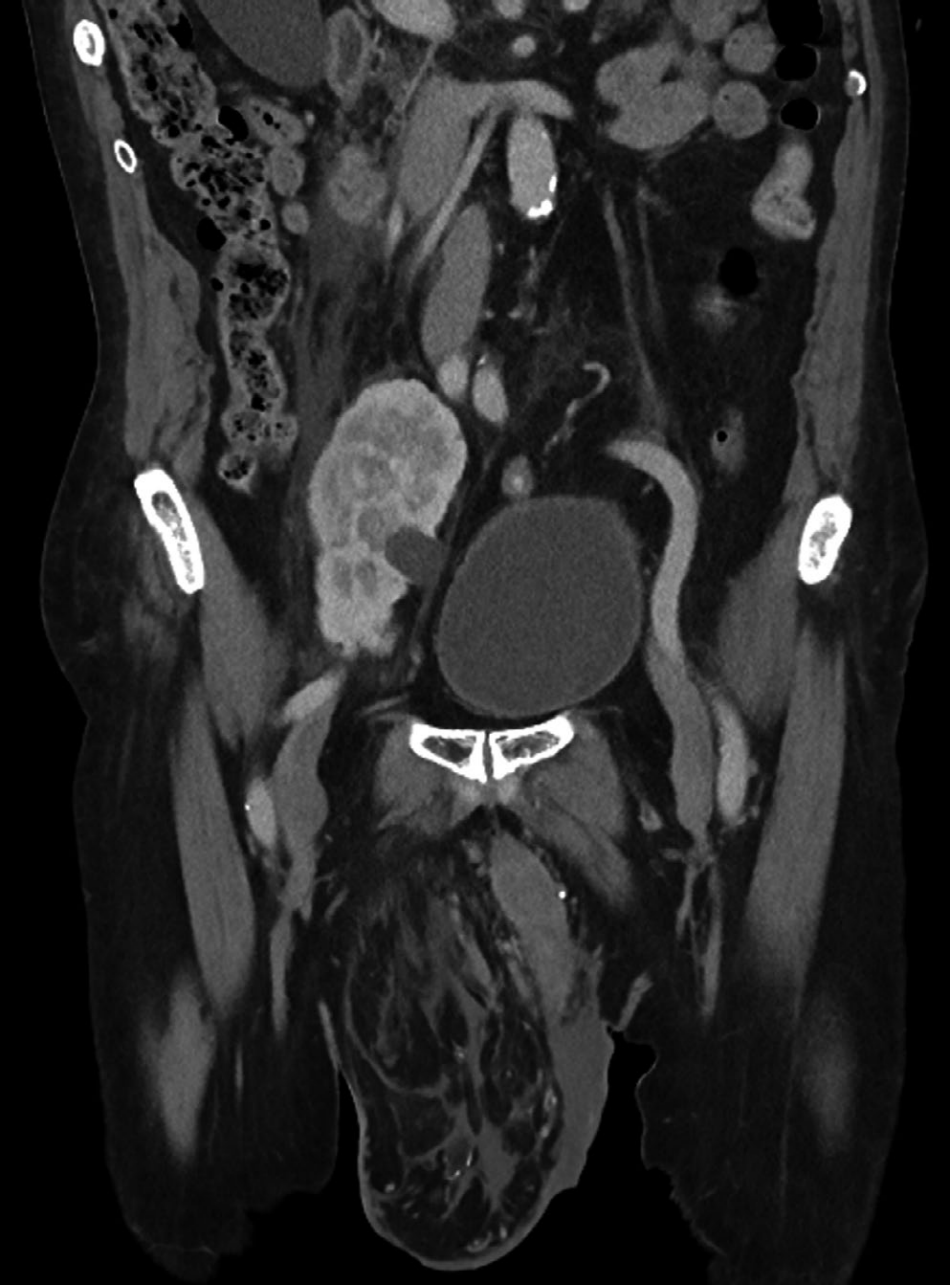
A 78‐y‐old gentleman was seen in the vascular surgery clinic due to peripheral arterial disease. Computed tomography discovered unconventional visceral arrangement. In this case, a large right inguinal hernia has distorted the usual retroperitoneal anatomy and migrated the right kidney to the right iliac fossa, approaching the hernia neck. The right kidney exhibits a degree of hydronephrosis

A renal transplant, accompanied by an overlying Rutherford‐Morrison incision, is preferentially anastomosed within the right iliac fossa (RIF) due to relative ease of access to the iliac vessels. Supernumerary pelvic kidney is rare and more commonly seen on the left. It results from abnormal division of the metanephric cord and can be differentiated from duplex kidney by an independent blood supply.^1^, ^2^ In this image (Figure 1), a large inguinal hernia has distorted conventional anatomy and the right kidney has descended into the RIF and hernia neck. In this case, the renal hilum is facing anteromedially with a degree of hydronephrosis.

## CONFLICT OF INTEREST

DD and LW have no conflicts of interest to declare.

## AUTHOR CONTRIBUTIONS

DD and LW: conceived the idea for the article. DD: wrote the original article and final version was reviewed by LW.

## ETHICAL APPROVAL

Written consent has been provided by the patient for publication of this image. No ethical approval was required for this work.
